# Kansas and Illinois

**Published:** 1886-06

**Authors:** 


### KANSAS AND ILLINOIS.

There may be a few opinionated eastern dentists who believe that their brethren of the west are as much behind them in professional attainments as they are upon the dial of the clock; that he of the western frontier still uses the turnkey for extraction, and is unacquainted with the dental engine and the electric mallet. If such ignorant ones there be, we can only wish that for their own edification they had been present at the late meeting of the Kansas State Dental Society. A more earnest and intelligent body of professional workers it has not been our good fortune to meet. Men who are so ardent in study and investigation that, after ten o'clock of the third session of the day, they will listen for more than an hour to a technical address, and not a single one of them betray a sign of weariness, are not found in every dental society.

The Kansas State Dental Society meeting for 1886, held at Topeka, the State capital, was a grand success. Not only was the attendance large, but the papers read were good, and the discussions fervent and intelligent. The clinics were a marked feature, and some of them were unique. It was the first time that we ever saw the whole process of making a full gold plate, from the refining of the gold to the final finishing, demonstrated before a dental society. Yet this was done at the Kansas State meeting, by Dr. Howe, of Lawrence, and the editor of this journal has the plate so made, to exhibit as a specimen of western dentistry. Great credit for the result of the meeting is due to the enthusiastic, generous souls who so freely spent their time and money to make of the meeting a success. From the distinguished President, Hon. L. C. Wasson, of Ottawa, down to the latest admitted member, all put forth every effort to add to the interest of the occasion. An excellent State dental law aids materially in elevating the professional status of the State. All hail, Kansas! Though one of the youngest in our legislative family of States, she is growing professionally with a rapidity characteristic of her wondrous soil.

The Illinois State meeting, held at Rock Island the week succeeding the Kansas meeting, was a very profitable one. In none of the States is there a State society whose transactions are read with more interest than in Illinois. In no State or local society meeting that we have ever attended was there such a grappling for basal principles as in Illinois. Long-winded "incidents of practice"

stories, in which the whole pedigree of the patient is related with minutest exactness, are not tolerated in this society.

A remarkable exhibition, and one that, we think, is entirely without precedent in society meetings, was that of Prof. G. V. Black, who had his incubator and sterilizing apparatus with him, and who made daily exhibitions of cultures of micro-organisms and demonstrated the changes wrought by them. In depth of research and breadth of the scope of true scientific investigation, the Illinois State society is not surpassed by any organization in this country. We can only refer our readers to the report of the meeting in this journal, and assure them that they will find plenty of food for thought therein.

